# Self-Enhancement and the Medial Prefrontal Cortex: The Convergence of Clinical and Experimental Findings

**DOI:** 10.3390/brainsci12081103

**Published:** 2022-08-19

**Authors:** Saeed Yasin, Anjel Fierst, Harper Keenan, Amelia Knapp, Katrina Gallione, Tessa Westlund, Sydney Kirschner, Sahana Vaidya, Christina Qiu, Audrey Rougebec, Elodie Morss, Jack Lebiedzinski, Maya Dejean, Julian Paul Keenan

**Affiliations:** 1 Department of Biology, Montclair State University, 320 Science Hall, Montclair, NJ 07043, USA; 2 Cognitive Neuroimaging Laboratory, Department of Biology, Montclair State University, 320 Science Hall, Montclair, NJ 07043, USA

**Keywords:** frontal cortex, PFC, self-enhancement, self-deception, SE

## Abstract

Self-enhancement (SE) is often overlooked as a fundamental cognitive ability mediated via the Prefrontal Cortex (PFC). Here, we present research that establishes the relationship between the PFC, SE, and the potential evolved beneficial mechanisms. Specifically, we believe there is now enough evidence to speculate that SE exists to provide significant benefits and should be considered a normal aspect of the self. Whatever the metabolic or social cost, the upside of SE is great enough that it is a core and fundamental psychological construct. Furthermore, though entirely theoretical, we suggest that a critical reason the PFC has evolved so significantly in *Homo sapiens* is to, in part, sustain SE. We, therefore, elaborate on its proximate and ultimate mechanisms.

## 1. The Prefrontal Cortex and Self-Enhancement

The Prefrontal Cortex (PFC) mediates or is involved in self-awareness, language, inhibition, planning, and abstraction, whilst also interacting with most other areas of the brain directly or indirectly [1,2]. The primary function of the PFC centers around executive functions, for which it has obtained the moniker of “central executive” [3]. Furthermore, the PFC’s subdivisions (e.g., the dorsal and medial PFC, the medial orbital frontal cortex, etc.) act as functionally specific processors that can operate and interact with one another [4,5], which, in turn, influences other cortical and non-cortical regions. Another prominent function of the PFC, at least in humans, is a “first-person-evaluator” [6], which refers to its ability to allow humans to develop and maintain a sense of self [7,8,9,10,11,12,13]. This function of the PFC dates to the early beginnings of neuroscience [14,15]. Alongside these significant processes, one must emphasize the PFC’s equally essential functions, such as memory [16,17], modeling outcomes [18], abstract thinking [19,20], emotion [21], cognitive control of internal goals [22,23], and language processing [24].

Here, we suggest that self-enhancement (SE) stands as a critical underlying factor as to why the PFC might have evolved so dramatically in humans. Self-enhancement is described as the tendency to unrealistically perceive one’s image of oneself in a positive direction [25,26,27]. When one self-enhances, they typically exaggerate their strengths and downplay their weaknesses. While the evolutionary advantages of other frontal cortex functions are immediately obvious (e.g., abstract thinking, planning, and emotional regulation), SE’s contribution may be less noticeable. Through analyzing how SE occurs, how the PFC is responsible for it, and its potential evolutionary purpose, we hypothesize that the PFC evolved, in part, to develop SE.

Self-enhancement refers to the tendency to maintain an often unrealistic, positive view of the self [25,27]. In order to maintain this tendency, SE creates a false self-perception where one makes judgments about oneself that are ungrounded in reality [28]. Self-enhancement is typically defined as something that occurs continuously, meaning when one produces an unrealistic, positive view of the self, it is maintained for an extended period [29] and extends across all dimensions of cognition, including exaggerating potential success in the future [30], only acknowledging positive feedback [31,32], falsely reporting higher test scores [33], and overestimating social approval [34].

The evidence that SE is mediated via the PFC is not simply correlational, as Transcranial Magnetic Stimulation (TMS) studies have produced direct evidence for the involvement of the medial PFC, or MPFC [35]. This has been shown by demonstrating that stimulation of the MPFC reduces the tendency to self-enhance [36,37]. More specifically, disrupting the MPFC while participants were rating themselves or their best friend caused them to perceive themselves as less “enhanced” compared with no disruption of the MPFC. These studies demonstrate a causal link between the MPFC and SE, as “virtual” removal of the MPFC leads to a reduction in SE.

Self-enhancement appears to be mediated through an accumulation of processes centered within the PFC, which is not surprising given its rich interconnected nature [4,5,38] and its many functions, such as pain processing [5], memory formation [39], creativity [40], etc. Among the vast array of functions of the PFC, some appear to be more directly involved with SE, including memory retrieval [41], conscious deliberation [42], morality [43], emotion regulation [2,3], and self-evaluation [44,45,46].

The PFC is involved in autobiographical (i.e., episodic, first-person) memory recall [47] and the recollection of self-relevant information [48]. During the retrieval process, the PFC often places an emotional value to autobiographical memories [49]. Lin et al. observed this in a typical fMRI design [41]. Scans were taken while participants took part in autobiographical memory recall tasks, where they would recall an autobiographical memory and evaluate it emotionally. Their analysis revealed the presence of blood oxygen level-dependent signals in the ventromedial PFC (vmPFC) during the retrieval of the memory. These signals would modulate depending upon the emotional intensity, therefore, correlating with the emotional intensity of the memory. These findings suggested that the vmPFC processes self-relevant information and is involved in associating emotional values with autobiographical memories. The extent of complexity that the value has is unknown, but it has been observed that during activation of the vmPFC, memories can be associated with simple values, such as “liked” and “positive” or “disliked” and “negative” [39,41]. Due to the SE involving the creation of illusory realities, this function is likely essential in order for SE to occur. Self-enhancement could involve the changing of a previous event from a “disliked” memory to a “liked” memory or vice-versa. This simple value change could lead to a completely different outlook on a previous event, regardless of the reality it holds.

## 2. Consciousness, Morality, Self, and Self-Enhancement

Conscious deliberation is a process where one forms a perspective and prediction of the future based upon (typically) past, present, and future considerations [42]. The neural network most involved in this process consists of the vmPFC, medial temporal lobe, and medial posterior regions, which are commonly considered the default mode network [50,51]. Among the numerous brain areas involved in this neural network, the vmPFC plays an essential role by mentally simulating events in the future [52,53]. Mentally simulating the future allows individuals to self-enhance, and this is typically correlated with activity in the PFC [54,55]. As noted previously, some individuals deem the occurrence of positive future events as far more likely to occur than negative ones [56]. Furthermore, most individuals demonstrate a higher probability to self-enhance when speculating upon events that are relevant to personal goals [57] and focus on short-term consequences relating to themselves [58]. Research for how the vmPFC causes SE to occur is still ongoing. However, studies have suggested possible mechanisms. The vmPFC has been found to modulate mental simulations of future events by modulating the associated emotional valence, making the intensities of emotions invoked by the mental simulations either more or less intense [57]. The vmPFC does this with both near and future events through the activation of different sections of itself [59,60,61]. Alongside conscious deliberation and memory retrieval, the PFC also accomplishes SE during the development of morality via manipulating emotional context.

Moral decision-making is the evaluation of actions while considering established norms and values [62]. The moral decision-making process is often a conscious and effortful task [63]. Through interacting with other brain networks, such as the temporal lobes and subcortical limbic structures [64], the PFC allows for moral decision-making [65]. Moreover, the PFC can change the desirability of moral decisions through these interactions, alongside interactions with the striatum [43]. This leads to the development of morality, such that if an individual views an action as desirable, they will associate it with being morally right to avoid the psychological repercussions and potential conflicts [29,66]. This process, we believe, is often the basis for SE. Due to the power of SE, one can create an illusion of reality by convincing themselves that an action is morally right when, logically, it is wrong. By continuously desiring to perform morally wrong actions and repetitively associating them with being morally right, one can self-enhance, regardless of any actuality.

SE can result in numerous nuances in moral decision-making. In addition, the principles of utilitarianism and deontology are of interest when discussing our current argument about moral decision-making, and it can be explained through the trolley problem. A viewpoint from a utilitarian perspective would support that killing a loved one over a group of people is morally correct, as the positives outweigh the negatives. However, a viewpoint from a deontological perspective implies that both choices are morally incorrect, as purposefully harming others is unacceptable, regardless of the situation. Regarding previous statements, when activity in the MPFC is increased, it causes the affected person to feel more conscious of their decisions and the impact they will make. This, in turn, makes them more likely to perform SE, which is based on the idea that the PFC can change the desirability of certain actions; thus, an individual will see a certain action as desirable and associate it with being morally correct. When applying this to the trolley problem, saving a loved one over a group of unknown people appears more desirable. Therefore, the individual will SE and create a false reality, in which it seems they made the morally correct decision, even though it is logically wrong, to avoid conflict and psychological repercussions.

Persons with Narcissistic Personality Disorder (NPD) have excessive SE [67]. Typically, one’s emotions will fluctuate depending on events within their everyday life. This is demonstrated in studies examining how emotions are altered depending upon a person’s inclusion or exclusion from social groups [68]. Individuals with NPD have exhibited the ability to consistently and sturdily self-enhance their emotions during moments that were meant to invoke insecurity, causing them to feel positive emotions, such as grandiosity and high self-esteem [67,69]. These persons appear to use SE as a defensive measure against negative emotions, such as humiliation or shame, causing further SE by associating themselves with positive characteristics, such as “thick-skinned” [70,71]. This ability to self-enhance to avoid negative emotions, as well as increase positive ones in a similar fashion, has been seen in healthy individuals as well, albeit to a lesser extent [25,72]. NPD is associated with excessive activity in the PFC [73,74], and disruption of the PFC via TMS appears to decrease the degree of sub-clinical NPD an individual may possess [75].

Self-evaluation is a conscious process, whereby a decision is made regarding oneself [76]. The medial PFC (MPFC) mediates the conscious processes associated with self-evaluations [44,45,46]. More specifically, it has been suggested that the MPFC plays a role in allowing the consciousness to access self-knowledge [77,78,79]. Alongside this, the MPFC associates mental states or perspectives when accessing self-knowledge [78,80]. Through both performing self-evaluations and associating mental states with self-knowledge, the MPFC creates illusory realities regarding the present and past self [81]. Specifically, through associating unrealistic perspectives with the present or past self, one may create an objectively false self-image. This method of SE can be exhibited even by healthy individuals, causing them to associate with overly positive characteristics when they, in fact, lack those traits [82]. Such behavior plays a vital role in making individuals overconfident during task performance. Since individuals are able to self-enhance when considering one’s self-knowledge, individuals can self-enhance their self-perceived abilities [82,83]. As a result, individuals appear to develop overconfidence biases [84], where they believe they can perform better than their objective skills allow them to [85]. These changes in self-evaluation have also been found to boost implicit self-esteem, which affects how individuals evaluate objects that are relevant to their identity [86]. For example, people have been found to inflate the monetary value of their property [87], view individuals who are similar to them as more attractive [86], and view individuals within their social group more positively [34,88,89].

In a society where one’s intelligence is valued, it is not surprising that SE is seen in persons reporting what they “know”. This behavior of overclaiming can be isolated to the PFC using a word knowledge test. Participants were randomly presented with a list of words and asked if they knew the definitions. Unbeknownst to the participants, 50% of the words were fake, and, thus, claiming knowledge of these words was impossible. Without TMS and under sham conditions, overclaiming occurred at a significant rate. However, following MPFC TMS overclaiming was reduced [90]. The role of the MPFC in overclaiming appears to expand under conditions of social pressure [91], which implies that overclaiming via the PFC likely exists to give one a social advantage. This makes sense, as overclaiming knowledge can lead to personal gains [92,93,94,95].

## 3. The Costs and Benefits of Self-Enhancement

However, not surprisingly, the cost–benefit ratio of SE has been debated. Several clinical and social-personality psychologists have argued that SE is maladaptive, listing several indicators [96,97,98]. Some psychologists believe that SE could cause individuals to harm their interpersonal relationships, as SE can lead to making inappropriate and excessive demands of others [99], not acknowledging suffering in work and love lives [100], and alienating themselves from others due to the belief they are above others [101]. Moreover, it is possible that SE could also cause damage to the self by causing individuals to lose their sense of personal identity [102], never reach self-actualization, and face frequent failure due to the belief that they can accomplish insurmountable tasks [103]. These shortcomings could all be argued to stem from one foregoing self-adjustment and, instead, undergoing SE [29]. In other words, instead of admitting to a fault and fixing one’s flaws, one can simply create an illusory self-perception and rid themselves of the psychological pressure. Trivers has, in fact, elegantly laid out the costs and benefits of SE (a factor of self-deception) and argued that the increase in confidence provides performance and social benefits, particularly the ability to both become a better deceiver and a more convincing persuader [104]. These theories were tested through the manipulation of participants’ overconfidence. Increases in overconfidence lead to an increase in persuasiveness, which the authors directly tied to an evolved SE cognitive architecture [105].

In contrast, previous studies have demonstrated that self-enhancers will typically be perceived more positively by others. This may be due to the fact that self-enhancers have reduced illusory social constraints and form stronger social bonds [106]. Self-enhancers have more extensive social networks, are more associated with leadership behaviors by peers, and experience greater daily contact with loved ones [107]. In a series of experiments examining the relationship between overconfidence and status, it was found that overconfidence would lead to individuals enjoying a higher status in both short-term and long-term groups. Additionally, it was found that overconfidence would make an individual appear more competent to others. Likewise, self-enhancers have been discovered to be perceived as more physically attractive, as shown by Holtzman and Shrube [108] when they found a positive narcissism–attractiveness correlation. Alongside receiving benefits in their perception, self-enhancers have also been found to experience benefits in task performance. O’Mara and Gaertner found that self-enhancers are more confident in performing tasks and, therefore, perform them better [109]. They asked two groups to perform a creative task, but only allowed one group to perform SE prior to the task (they were instructed to exaggerate their creativity in comparison to others). They found that if the participants self-enhance, then participants perform better at creative tasks, such as listing the uses of mundane objects. In terms of further benefits, it appears that being able to self-deceive and self-enhance can provide individuals with a better ability to deceive and enhance others [104].

It has been argued that without SE, individuals would be more susceptible to depression of mood, becoming unmotivated, being negative, etc. [27]. Many individuals, when sad or depressed, will undergo personal adjustment to promote themselves to feel happy [101,110]. Though clearly over-simplified, there are often two ways to undergo personal adjustment, which is either through SE or real-life gains [27,29]. Through pursuing actual benefits, one could attain their goals and accomplish personal satisfaction in a physical (if any) and psychological fashion. Nevertheless, through SE, one can forgo the effort of accomplishing goals and receive psychological satisfaction. Psychologists have argued that going for SE instead of real-life gains can cause serious long-term harm if long-term problems are not solved [101,103]. For example, when individuals were asked to evaluate their academic ability and had their academic abilities tested, individuals who self-enhanced when evaluating their academic abilities appeared to become less motivated and disengaged from academics over time [103]. This disengagement is most likely due to individuals not acknowledging their shortcomings and attempting to better themselves.

SE apparently serves as a buffer against adversity within one’s environment [111]. Distorting reality can help face the harshness and negativity of life’s curveballs that are near impossible to fix. Being in a stressed or depressed state causes individuals to use more energy than usual, disrupting normal metabolic pathways within the brain, accelerating cell injury, and causing unnecessary immune system responses [112]. As the brain is already a voracious consumer of oxygen and sugar [113], it would be disadvantageous for even more energy to be used towards stress and depression. An example of this would be if someone caused a fire by leaving the stove on and, consequently, lost their favorite pet to it. One could go through all the past events that potentially caused the incident. As a result, one would use an exuberant amount of psychological and physical effort to consider what one could have done differently. On the other hand, one could also simply distort their own reality and state that it was not their fault. We have found that affect and self-enhancement are directly tied together in regions of the PFC, as determined via TMS [114]. This suggests at least some degree of mood enhancement, SE, and the PFC. This clearly makes sense as self, emotion, and the PFC are highly related [115,116,117,118,119,120].

Individuals self-enhance to avoid the adversities they face from societal pressures [29,121,122]. The self-centrality principle states that “self-centrality breeds self-enhancement”, or in other words, individuals typically self-enhance the most on traits that they consider central to their self-image [26]. Gebauer et al. (2017) tested this principle using three sets of studies to examine how the self-centrality principle applies to Christian populations. The results provided consistent evidence for the self-centrality principle, discovering that Christians self-enhanced more than non-believers in characteristics that reflected on core Christian beliefs. These characteristics included their knowledge of the different sects of Christianity, knowledge of communion, knowledge of agency, understanding of the commandments of faith, and understanding of the commandments of communion [26]. Most likely, if these traits were left not self-enhanced, the individuals would face psychological stress, as they would label themselves as ignorant or uninformed about the religion they believe in. SE, therefore, allowed them to avoid this stress. This principle also extends to cultural pressures [123,124,125]. Previous studies have shown that individuals self-enhance differently if they are from different cultural backgrounds [126,127,128]. East Asians and Asian Americans have shown fewer signs of self-serving bias when compared to Westerners [129]. Individuals from India have displayed higher levels of optimism than others when predicting the outcomes of negative events [127]. These differences have been attributed to the divergence of collectivist and individualistic cultures, meaning cultures that give more priority to their perceived “group” and cultures that give more priority to the individual, respectively [130]. Other studies suggest that these differences are attributable to cultural differences in modesty and the way it plays a role in societal pressure [131]. Again, individuals differentially modulate what they self-enhance based upon different societal pressures in order to avoid stress and adversity. It is important to note, however, that studying cultural differences in self-enhancement is very difficult and ever changing. This is due to the views of different cultures being heavily affected by the personal biases and experiences of researchers, causing misinterpretations of data [132,133].

## 4. A New Role for the Prefrontal Cortex

The argument that SE is used as a method to reduce and prevent depression provides a new fundamental role for the PFC. This view is supported by recent data collected by Duran et al., showing how the disruption of regions associated with self-enhancement leads to a significant decrease in mood [114]. Specifically, replicating the parameters of Kwan et al.’s study, it was found that TMS decreased mood following MPFC TMS. These data are comparable to the observations in some clinical populations where MDD is associated with a lack of self-enhancement [114]. Furthermore, social anxiety disorder (SAD) and the self are intimately related. Using behavioral measures, it was first found that the SAD group reported significantly greater embarrassment for self-faced images than the controls. Employing fMRI, the SAD group demonstrated enhanced self-related activation in the left PFC compared to the controls. Interestingly, there was a positive correlation between the self-related activity and the Liebowitz Social Anxiety Scale in the MPFC [134].

**Figure 1:**
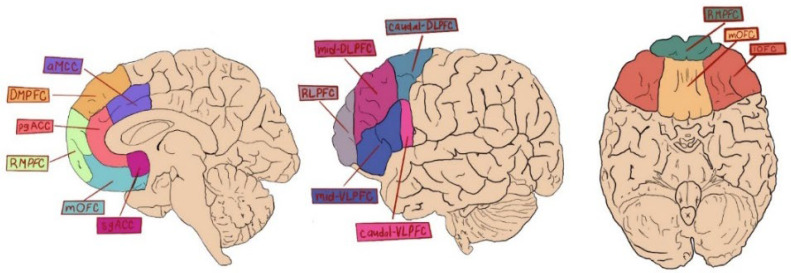
The PFC includes numerous subdivisions. The basic divisions are noted here in medial sagittal, lateral sagittal, and axial orientations. While there is not enough research on SE to draw firm delineations, here, we note differences that may exist. The rmPFC is involved in impression management [137], which may include the general SE abilities of the medial PFC [91,137,138,139]. The ventral PFC regions (sgACC, mOFC, and VLPFC) are involved in social SE [140,141]. The lateral regions of the PFC (DLPFCVLPFC) are involved in long-term SE of the core self [9].

We suggest, here, that the importance of SE is as a reductive and preventative “medicine” against depression, alongside its ability to save psychological and physical energy. This provides the argument that the PFC evolved, in part, to allow for SE to occur. The evolutionary advantages of SE touch on many realms, including task-management, social perception, and one’s own self-concept (Figure 1). It is interesting to consider that hallucinogenic drugs are now being employed in the treatment of numerous disorders, including Major Depressive Disorder [135], and, as such, both the nature of reality and “the true self” are serious topics of scientific inquiry [136].

The statement that self-enhancement provides a positive psychological outlook is a claim that opposes many notions of mental health; therefore, it should be looked at with skepticism. A recent meta-analysis did just this [29]. The study examined more than 125,000 participants and found a direct positive correlation; as self-enhancement increased, so did successful, healthy personal adjustments. Further, the relationship seemed causal (i.e., self-enhancement causes a positive affect), as determined by longitudinal variables. While there were some negative social correlations, the overall impact of self-enhancement was strong and robust. This study, by far the largest on the topic, does not inform us about neurological correlates or if natural selection plays any role in self-enhancement. However, it does support the notion that perhaps the PFC serves as an anti-depressant buffer through altering reality.
